# Shared expression of phenotypic markers in systemic sclerosis indicates a convergence of pericytes and fibroblasts to a myofibroblast lineage in fibrosis

**DOI:** 10.1186/ar1790

**Published:** 2005-07-21

**Authors:** Vineeth S Rajkumar, Kevin Howell, Katalin Csiszar, Christopher P Denton, Carol M Black, David J Abraham

**Affiliations:** 1 Centre for Rheumatology & Connective Tissue Disease, Department of Medicine, Royal Free Campus, University College London, London, UK; 2 Cardiovascular Research Center, John A Burns School of Medicine, University of Hawaii, Honolulu, HI, USA

## Abstract

The mechanisms by which microvascular damage leads to dermal fibrosis in diffuse cutaneous systemic sclerosis (dcSSc) are unclear. We hypothesized that microvascular pericytes constitute a cellular link between microvascular damage and fibrosis by transdifferentiating into myofibroblasts. We used a combination of immunohistochemistry and double immunofluorescence labelling of frozen skin biopsies taken from normal and dcSSc patients to determine whether a phenotypic link between pericytes and myofibroblasts exists in dcSSc. Using α-smooth muscle actin, the ED-A splice variant of fibronectin (ED-A FN) and Thy-1 to identify myofibroblasts, we demonstrated the presence of myofibroblasts in fibrotic dcSSc skin. Myofibroblasts were totally absent from control skin, atrophic stage dcSSc skin and non-lesional skin. Using double immunofluorescence labelling, both myofibroblasts and pericytes were shown to express ED-A FN and Thy-1 in dcSSc skin but not in control skin. Proliferating cell nuclear antigen was also expressed by myofibroblasts and pericytes in dcSSc skin while being absent in control skin. These observations suggest that the presence of myofibroblasts may represent a transitional phase during the fibrotic stages of dcSSc and that Thy-1^+ve ^pericytes participate in the fibrogenic development of dcSSc by synthesizing ED-A FN, which may be associated with a proliferation and transition of pericytes and fibroblasts to myofibroblasts, thus linking microvascular damage and fibrosis.

## Introduction

Systemic sclerosis represents a spectrum of connective tissue disorders, characterized by chronic and debilitating fibrosis of the skin and internal organs, most notably the lungs, kidney, cardiovascular system and gastrointestinal tract [1]. While the pathological endpoint of diffuse cutaneous systemic sclerosis (dcSSc) is recognized as clinical fibrosis, the origins are thought to lie in the microvasculature, as over 90% of patients exhibit chronic microvascular damage prior to the onset of clinical fibrosis [2]. Beyond that, however, very little is known about the cellular and molecular mechanisms that produce chronic fibrotic lesions in dcSSc. Microvessels comprise two cell types, endothelial cells and pericytes. Analyses of microvascular changes in dcSSc have focussed almost solely on the contribution of endothelial cells, largely overlooking the potential role of pericytes. Pericytes reside at the abluminal surface of microvessels and are in intimate contact with the underlying endothelium through numerous points of cell-cell contact. It has become increasingly clear that pericytes are vital in maintaining normal vascular homeostasis and regulating vascular phenotype in disease [3]. Given their central role in modulating endothelial cell function, it is clear that the pronounced changes observed in endothelial cells during dcSSc will also alter pericyte phenotype and function. Consistent with this idea, we have previously demonstrated that microvascular pericytes become activated and express platelet-derived growth factor-beta (PDGF-β) receptors in dcSSc, a phenotype not seen in normal skin [4].

Of potential significance in fibrotic diseases is the phenotypic similarity between pericytes and myofibroblasts. Like pericytes, myofibroblasts express alpha smooth muscle actin (α-SMA) and are strongly associated with fibrotic tissue [5]. Originally described in wound tissue, the primary role of myofibroblasts is contraction of early granulation tissue [6]. After wound contraction, myofibroblasts are believed to be removed by apoptosis, a crucial step in wound resolution [7]. Failure of the local myofibroblast population to undergo apoptosis has been postulated as a mechanism whereby an acute wound response can become a chronic fibrotic disorder [8]. Differentiated myofibroblasts can be distinguished from normal fibroblasts by the expression of α-SMA and the ED-A splice variant of fibronectin (ED-A FN). ED-A FN expression precedes the appearance of α-SMA-positive myofibroblasts and is considered a crucial factor in promoting the formation of myofibroblasts [9]. Blocking the interaction between ED-A FN and the cell surface *in vitro *inhibits the transforming growth factor-beta (TGF-β)-mediated induction of α-SMA synthesis and resultant myofibroblast formation. Thus, the *de novo *synthesis of ED-A FN appears to be a pre-requisite of α-SMA expression and myofibroblast differentiation [10]. Increased expression of ED-A FN has been reported in other fibrotic disorders [11,12], however, not in dcSSc. In common with practically all fibrocontractive diseases, the presence of myofibroblasts has been described in dcSSc skin [13,14], however, beyond that very little is known about their precise role in the disease process. For example, the mechanisms of their appearance and persistence within fibrotic tissue remain unclear, as does their contribution to increased matrix deposition.

Another factor implicated in the differentiation of myofibroblasts is Thy-1, a cell surface glycoprotein, which is differentially expressed by fibroblasts [15]. Thy-1^+ve ^and Thy-1^-ve ^populations of fibroblasts are known to be functionally distinct with regards to production of cytokines and extracellular matrix [16,17] and it was recently demonstrated that only Thy-1^+ve ^fibroblasts are capable of differentiating into myofibroblasts after treatment with TGF-β [18], suggesting that Thy-1 is a marker of cells with myofibroblastic potential.

In liver fibrosis and glomerular fibrosis, pericytes have been proposed as a source of myofibroblasts [19,20]. This hypothesis is compatible with the clinical picture in dcSSc of chronic microvascular damage followed by fibrosis. It is known that pericytes have the capacity to act as precursor cells for other differentiated mesenchymal cells [21], including collagen-synthesizing fibroblasts [22,23]. Therefore, we hypothesized that microvascular pericytes are precursor cells for myofibroblasts in dcSSc skin. Using double immunofluorescence labelling, we have been able to show that pericytes and myofibroblasts share an identical phenotype with regards to α-SMA, ED-A FN and Thy-1 in dcSSc skin.

## Materials and methods

### Patient and biopsy specimens

All patients in the study were diagnosed as having diffuse scleroderma (*n *= 16) using the classification established by LeRoy *et al. *[24]. The SSc cohort included 10 patients with fibrotic dcSSc and six patients with atrophic dcSSc. Following informed consent and ethical approval, lesional skin was taken from the forearms of patients with fibrotic scleroderma and non-lesional skin was taken from the lower back. Non-lesional skin was defined as having a modified Rodnan skin score of zero. Site-matched normal skin samples were obtained from sex- and age-matched volunteers (*n *= 8). Clinical characteristics are presented in Table 1. Disease severity and internal organ involvement was assessed according to the recently published consensus for SSc studies [25]. Therefore, skin involvement was assessed using the modified Rodnan skin score and gastrointestinal involvement was defined symptomatically. A restrictive pattern of pulmonary function abnormalities with reduction in forced vital capacity and diffusion capacity for carbon monoxide below 80% of predicted value (based on age, sex, height and ethnic origin) was used to assess interstitial lung involvement. This was confirmed by high-resolution computed tomography of the chest. Diagnosis of pulmonary arterial hypertension was confirmed by right heart catheterization. Cardiac involvement was considered present if any significant conduction defects were found on electrocardiogram or impaired left ventricular function, or if haemodynamically significant pericardial effusion was detected by echocardiography. A greater than four-fold elevation of creatinine kinase accompanied by the clinical finding of proximal weakness defined muscular involvement, whilst renal involvement was determined by history of scleroderma renal crisis or significant impairment in creatinine clearance (<65 ml/min) without alternative explanation.

All biopsies were embedded in OCT (optimum cutting temperature compound) and immediately snap frozen in isopentane cooled by liquid nitrogen and subsequently stored at -70°C prior to cryosectioning.

### Antibodies

Microvascular pericytes were identified using 1A4 (Sigma, UK), a mouse monoclonal antibody against α-SMA [26]. The monoclonal antibody AS02 (Oncogene, UK) was used to identify Thy-1 [27] and the PAL-E monoclonal antibody (Uden, Holland) recognizes endothelial cells with high sensitivity and specificity [28,29]. ED-A FN was identified using the 3E2 monoclonal IgM antibody (Sigma) [30] and a rabbit polyclonal antibody recognizing lysyl oxidase (LOX) was used to identify cells synthesizing collagen and elastin [31]. LOX plays a central role in catalysing collagen cross-linking within the extracellular matrix [32] and has been established as a surrogate marker for collagen-synthesizing cells [33]. Proliferating cells were labelled with a rabbit polyclonal antibody against proliferating cell nuclear antigen (PCNA) (Abcam, UK) [34]. Biotinylated secondary antibodies against mouse IgG and IgM and Vectastain ABC reagent were obtained from Vector Laboratories, (Peterborough, UK). All antibodies were diluted in PBS.

### Immunohistochemistry

Serial frozen sections (6 μm) were cut on a cryostat, air-dried and then stored at 80°C prior to use. Sections were fixed in ice-cold acetone and then blocked with normal horse serum and incubated with primary antibodies for 1 h at room temperature. Endogenous peroxidase was exhausted by incubation with H_2_O_2 _at room temperature for 15 mins in the dark. After washing, sections were incubated with the appropriate biotinylated secondary antibody (7.5 μg/ml) diluted in PBS for 30 mins, rinsed and then finally incubated with Vectastain ABC reagent for 30 mins (Vector Laboratories, Peterborough, UK). After washing, sections were visualized using 3-amino-9-ethylcarbazole and then washed in tap water, counterstained with haematoxylin and aqueously mounted with Crystal-Mount (Biomeda, CA, USA). Sections were viewed and photographed on a Zeiss Axioskop 2 mot plus microscope. Controls included an exchange of primary antibodies with isotype-matched control antibodies.

### Determination of PCNA-positive microvessels

In order to determine the proportion of microvessels expressing PCNA, serial cryosections were used. Briefly, serial cryosections were treated as above and stained for PAL-E and PCNA. Twenty fields of view were analysed using a ×20 Zeiss Plan-Neofluar lens and results were expressed as a percentage of PAL-E-positive vessels.

### Double immunofluorescence labelling

To investigate colocalization between cell-specific antigens, double immunofluorescence labelling was carried out. Briefly, cryosections were fixed in ice-cold acetone, blocked in serum and incubated with the first primary antibody for 1 h, rinsed and then incubated with the appropriate biotinylated secondary antibody (7.5 μg/ml) for 30 mins. Sections were rinsed and incubated with Avidin Texas Red 25 μg/ml for 30 mins. After blocking with serum, the sections were then incubated with the second primary antibody for 1 h, rinsed and incubated with an appropriate secondary IgG fluorescein (FITC) conjugate (12.5 μg/ml) for 30 mins. Sections were finally counterstained with 4,6-diamidino-2-phenylindole (DAPI) to visualize cell nuclei. The sections were then mounted using Gel-Mount anti-fade medium (Biomeda, CA, USA), and viewed using a Zeiss Axioskop 2 mot plus microscope with Axiovision software.

### Nailfold capillaroscopy

Nailfold capillaroscopy was performed using a Nikon optical system illuminated by a fibre optic light source. Images were analysed and recorded with a Hitachi CCD digital camera. Microvascular damage was analysed and quantified using the criteria established by Cutolo *et al*. Essentially, dcSSc patients were graded as having an early (E), active (A) or late (L) pattern of capillary damage [35].

### Correlation of immunohistochemistry with clinical findings

Patients were classified according to four immunohistochemical criteria:

1. evidence of myofibroblasts/ED-A FN,

2. evidence of collagen synthesis,

3. evidence of myofibroblasts/ED-A FN and collagen synthesis,

4. no evidence of either myofibroblasts/ED-A FN or collagen synthesis.

Disease duration, skin score and capillary damage were compared between groups. Statistical significance was determined by ANOVA and Fishers Exact test with *p *values <0.05 considered to be statistically significant.

## Results

### Myofibroblasts are present only in fibrotic dcSSc skin

**Figure 1:**
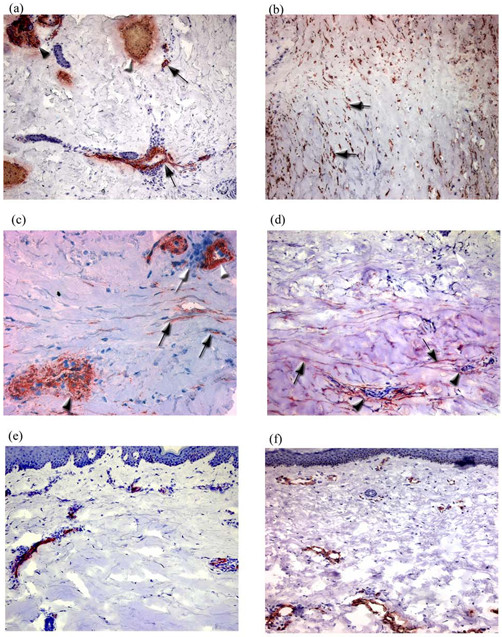
Detection of myofibroblasts in dcSSc skin. Cryosections from **(a) **normal and **(b-f) **dcSSc skin were stained with an antibody against α-SMA. In normal skin, α-SMA staining was restricted primarily to microvascular pericytes enveloping capillaries ((a) arrows), sweat glands ((a) black arrowhead) and smooth muscle cells of erector pili muscles ((a) white arrowhead). In dcSSc samples, α-SMA-expressing myofibroblasts were detected in the dermis ((b,c,d) black arrows). Myofibroblasts were predominantly detected in the lower reticular dermis of SSc skin ((c,d) black arrows) while interstitial cells in the papillary dermis did not express α-SMA ((c,d) white arrows). In reticular dermal layers, α-SMA staining was also detected in the perivascular region ((c,d) black arrowheads) while in the papillary dermal layers α-SMA immunostaining was restricted to microvessels ((c) white arrowhead). In (e) non-lesional and (f) late stage dcSSc, the distribution of α-SMA was similar to that seen in normal skin. Original magnification (a,b,e,f) ×10, and (c,d) × 20. α-SMA, alpha smooth muscle actin; dcSSc, diffuse cutaneous systemic sclerosis.

The distribution of myofibroblasts was investigated using the 1A4 monoclonal antibody against α-SMA. In normal skin, α-SMA immunostaining was predominantly restricted to microvascular pericytes, sweat glands and smooth muscle cells of the erector pili muscles (Fig. 1a). No α-SMA immunoreactivity was detected in interstitial fibroblasts (Fig. 1a). Six dcSSc cases were characterized by the presence of myofibroblasts (Fig. 1b). In five of these cases, myofibroblasts were located almost exclusively in the lower reticular dermis and were absent from the upper papillary dermis where α-SMA immunoreactivity was restricted to microvascular pericytes (Fig. 1c). In the remaining dcSSc case, myofibroblasts were detected in both the reticular and papillary dermis (data not shown). In reticular dermal areas containing myofibroblasts, α-SMA-expressing cells were also frequently observed in the immediate perivascular area (Fig 1c,d) while in the papillary dermis, α-SMA-expressing cells were only detected within the microvascular wall (Fig. 1c). Myofibroblasts were not detected in any of the non-lesional and atrophic dcSSc samples in which the pattern of α-SMA immunostaining was similar to that seen in normal skin (Fig. 1e,f).

### The presence of myofibroblasts correlates with the expression of ED-A FN but not collagen in dcSSc skin

**Figure 2:**
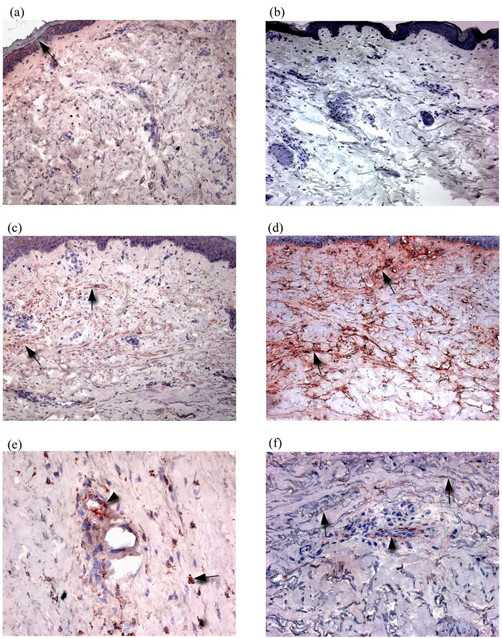
Increased expression of LOX and ED-A FN in dcSSc skin. Cryosections of **(a,b) **normal skin are compared with **(c-f) **dcSSc skin. In normal skin, immunostaining for LOX was detected in epidermal cells ((a) arrow). In dcSSc skin, immunostaining for LOX was detected in fibroblast-like cells throughout the dermis ((c,e) arrows) and in cells of the microvascular wall ((e) arrowhead). Little or no expression of ED-A FN was detectable in (b) normal skin, however, ED-A FN immunostaining was markedly increased in dcSSc skin ((d,f) arrows). Immunostaining for ED-A FN was also detected in cells of the microvascular wall ((f) arrowhead). Original magnification (a-d) × 10 and (e,f) × 20. dcSSc, diffuse cutaneous systemic sclerosis; ED-A FN, ED-A splice variant of fibronectin; LOX, lysyl oxidase.

Next we investigated whether myofibroblasts were associated with the presence of ED-A FN and collagen in dcSSc skin. Collagen-synthesizing cells were identified using an antibody against the enzyme lysyl oxidase (LOX) as previously reported [36,37]. In normal skin, expression of LOX was noted in cells within the epidermis and associated with collagen and elastic fibres in the dermis (Fig. 2a). In four dcSSc cases, an increase in LOX immunostaining was observed when compared with normal skin, principally in interstitial fibroblastic cells throughout the dermis (Fig. 2c) and cells associated with the microvasculature (Fig. 2e). Two of these dcSSc cases were also characterized by the presence of myofibroblasts. The distribution of LOX immunostaining in all atrophic dcSSc and non-lesional dcSSc tissue was similar to that seen in normal skin (data not shown).

**Figure 3:**
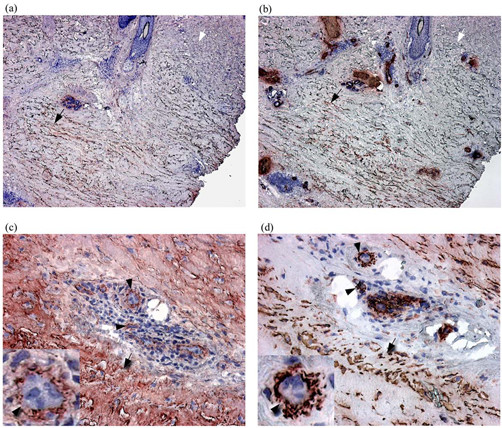
Expression of ED-A correlates specifically with myofibroblasts in dcSSc skin. **(a,c) **Serial cryosections were stained with antibodies against ED-A FN and **(b,d) **α-SMA. Both ED-A FN ((a,c) arrows) and α-SMA^+ve ^myofibroblasts ((b,d) arrows) were predominant in the lower reticular dermis of dcSSc skin. Note the absence of ED-A FN ((a) white arrow) and α-SMA^+ve ^myofibroblasts ((b) white arrow) in the papillary dermis. In addition, immunostaining for ED-A FN was also detected in the wall of microvessels ((c) inset, arrowheads) correspondingly containing α-SMA-expressing pericytes ((d) inset, arrowheads). Original magnification (a,b) × 10, (c,d) × 20, inset (c,d) × 40. α-SMA, alpha smooth muscle actin; dcSSc, diffuse cutaneous systemic sclerosis; ED-A FN, ED-A splice variant of fibronectin.

The distribution of ED-A FN was then evaluated. Little or no immunostaining for ED-A FN was detected in normal skin (Fig. 2b). However, in six dcSSc cases there was marked increase in ED-A FN staining, predominantly in fibroblastic cells and small capillaries (Fig. 2d,f). Significantly, increased ED-A FN deposition was only observed in those dcSSc samples containing myofibroblasts. ED-A FN is known to be a key mediator in the differentiation of myofibroblasts and, to our knowledge, this is the first report of increased ED-A FN in dcSSc skin. We then used serial cryosections to confirm that the expression of ED-A FN was localized to the presence of myofibroblasts. Increased immunostaining for ED-A FN was located predominantly in the reticular dermis mirroring the distribution of myofibroblasts (Fig. 3a,b). Papillary dermal layers, which were negative for myofibroblasts, contained little or no ED-A FN expression (Fig. 3a,b). In the lower reticular dermis in dcSSc, immunostaining for ED-A FN was also frequently observed associated with microvessels enveloped by α-SMA-positive pericytes (Fig. 3c,d).

### Increased dermal staining of Thy-1 in fibrotic dcSSc skin

**Figure 4:**
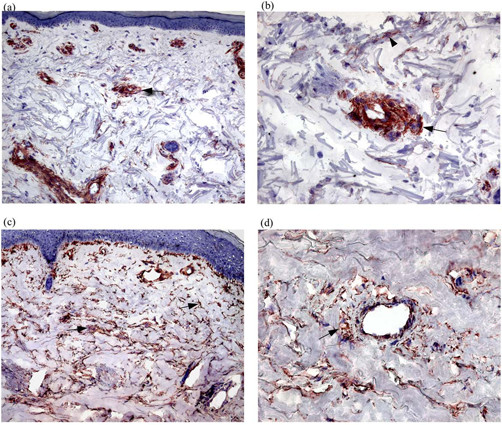
Expression of Thy-1 is increased in dcSSc skin. Cryosections from **(a,b) **normal and **(c,d) **dcSSc were stained for Thy-1 expression. In normal skin, immunostaining for Thy-1 was predominantly located within the microvascular wall and immediate perivascular region ((a,b) arrows). Thy-1 staining of interstitial fibroblasts was also detected ((b) arrowhead). In dcSSc skin, immunostaining of fibroblastic cells was considerably more pronounced throughout the interstitial dermis ((c) arrows) while perivascular immunostaining in dcSSc skin ((d) arrow) was less pronounced than that observed in normal skin ((b) arrow). dcSSc, diffuse cutaneous systemic sclerosis.

It was recently reported that myofibroblasts can only differentiate from Thy-1-expressing fibroblasts [18], therefore we analysed Thy-1 expression *in vivo *in order to identify putative sources of myofibroblasts. In normal skin, Thy-1 immunostaining was located predominantly in the microvascular wall and the immediate perivascular region (Fig. 4a,b). Occasional cells within the dermis were also positively stained in both the papillary and reticular dermal layers (Fig. 4b). In agreement with previous studies, no Thy-1 immunostaining was detected in the keratinocyte layers of the epidermis [38]. In all samples of dcSSc skin, there was a pronounced increase in Thy-1 staining throughout the dermis (Fig. 4c). In perivascular regions, Thy-1 immunostaining was frequently less pronounced than that observed in normal skin (Fig. 4d). All cases of atrophic dcSSc skin and non-lesional dcSSc skin showed a similar distribution of Thy-1 immunostaining to that observed in normal skin (data not shown).

### Microvascular pericytes express ED-A fibronectin and Thy-1 in dcSSc skin

As the observed immunostaining for Thy-1 was strongly associated with microvessels, we hypothesized that it may be in part attributable to expression by microvascular pericytes.

**Figure 5:**
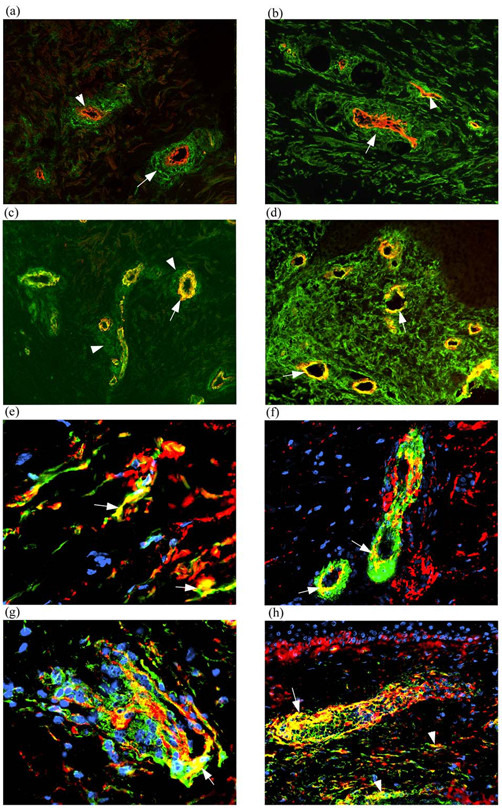
Double immunofluorescence labelling of normal and dcSSc skin biopsies. Cryosections from **(a,c) **normal and **(b,d) **dcSSc were double stained for endothelial cells using (a,b) PAL-E antibody and Thy-1 and (c,d) α-SMA and Thy-1. Thy-1 is labelled with FITC while PAL-E and α-SMA are labelled with Texas Red. In both (a) normal and (b) dcSSc, immunofluorescence for Thy-1 ((a,b) arrow, green colour) and PAL-E ((a,b) arrowhead, red colour) was consistently exclusive and showed no colocalization. In both (c) normal and (d) dcSSc, strong colocalization between Thy-1 and α-SMA was evident ((c,d) arrows, yellow colour). In normal skin, Thy-1 immunofluorescence that did not colocalize with α-SMA was observed immediately adjacent to microvessels ((c) arrowheads, green colour). Cryosections from dcSSc were double stained for **(e,f,g) **ED-A FN and α-SMA and **(h) **ED-A FN and Thy-1. ED-A FN is labelled with Texas Red while α-SMA and Thy-1 are labelled with FITC. Cell nuclei are counterstained blue with DAPI. Colocalization between α-SMA and ED-A FN was detected in dermal fibroblastic cells ((e) arrows, yellow colour) as well as in the microvascular wall ((f,g) arrows, yellow colour). Colocalization was also observed between ED-A FN and Thy-1 in both the microvascular wall ((h) arrow, yellow colour) and in dermal fibroblastic cells ((h) arrowheads, yellow colour). Original magnification (a-d,h) × 10, (e,f) × 20, (g) × 40. α-SMA, alpha smooth muscle actin; DAPI, 4,6-diamidino-2-phenylindole; dcSSc, diffuse cutaneous systemic sclerosis; ED-A FN, ED-A splice variant of fibronectin; FITC, fluorescein isothiocyanate.

Using immunofluorescence, we performed multiple labelling experiments of normal and dcSSc skin sections to simultaneously visualize endothelial cells, pericytes and Thy-1 immunopositive fibroblasts. Combinations of these markers are depicted in Fig. 5, highlighting the spatial relationship between Thy-1 immunofluorescence and the microvasculature. We and others have previously demonstrated that immunofluorescence staining for α-SMA and PAL-E, while being closely associated, do not colocalize, indicating that these markers can be used to discriminate between pericytes and endothelial cells [4,23]. When used in combination with the anti-endothelial cell antibody, PAL-E, immunofluorescence for Thy-1 and endothelial cells was separate and exclusive with no evidence that Thy-1 expression colocalized to endothelial cells in either normal or dcSSc skin (Fig. 5a,b). Conversely, Thy-1 immunofluorescence showed a marked colocalization with α-SMA expression by microvascular pericytes in normal (Fig. 5c) and dcSSc skin samples (Fig. 5d) confirming that the perivascular expression of Thy-1 could be attributed to pericytes. In normal skin, Thy-1 immunofluorescence that did not colocalize with α-SMA could also be detected immediately adjacent to small microvessels (Fig. 5c). We then carried out double-labelling experiments with antibodies against ED-A FN in combination with specific cellular markers to identify the sources of ED-A FN in SSc skin. Immunofluorescence for ED-A FN colocalized with interstitial α-SMA immunofluorescence in the dermis, confirming our serial immunohistochemical data that in dcSSc skin, myofibroblasts synthesize ED-A FN (Fig. 5e). Immunofluorescence for ED-A FN was found to colocalize with both Thy-1 and α-SMA within the microvascular wall leading to the conclusion that pericytes synthesize ED-A FN in dcSSc skin (Fig. 5f,g,h).

### Fibroblasts and pericytes show evidence of proliferation in dcSSc skin

**Figure 6:**
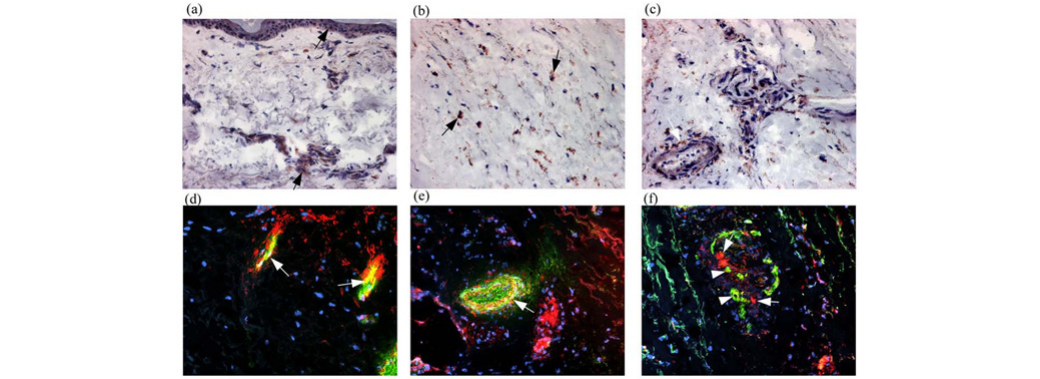
Distribution of proliferating cells in normal and dcSSc skin. Cryosections from **(a) **normal and **(b,c) **dcSSc were stained with an anti-PCNA antibody. In normal skin, PCNA immunostaining was restricted to cells within the epidermis and sweat glands ((a,b) arrows). In two out of ten dcSSc samples, PCNA was detected in fibroblastic cells ((b) arrows) and in microvessels ((c) arrows). Double immunofluorescence labelling of dcSSc skin: cryosections were double stained with a combination of antibodies against (d,e) PCNA and α-SMA and (f) PCNA and PAL-E. PCNA is labelled with Texas Red while α-SMA and PAL-E are labelled with FITC. Colocalization was detected with PCNA and α-SMA antibodies within the microvasculature ((d,e) arrows, yellow colour). When used in combination with PAL-E, PCNA-labelled cells ((f) arrows) were predominantly located adjacent and abluminal to endothelial cells ((f) arrowheads). Original magnification × 20. α-SMA, alpha smooth muscle actin; dcSSc, diffuse cutaneous systemic sclerosis; PCNA, proliferating cell nuclear antigen; FITC, fluorescein isothiocyanate.

In order to determine whether the appearance of myofibroblasts was accompanied by cell proliferation, we used an antibody against PCNA to analyse the distribution of proliferating cells in normal and dcSSc skin. In normal skin, PCNA immunostaining was detected in epidermal cells and cells associated with hair follicles and sweat glands (Fig. 6a). Little or no immunostaining for PCNA was seen in interstitial fibroblastic cells or microvessels. Analysis of the dcSSc samples revealed a marked expansion of PCNA immunostaining in two cases. PCNA staining was detected in dermal fibroblast-like cells (Fig. 6b) and was also evident within a proportion of microvessels (Fig. 6c). These two dcSSc samples were also characterized by the presence of myofibroblasts and increased ED-A FN expression. Double-labelling analysis with combinations of PCNA and α-SMA revealed colocalization between these proteins in a proportion of microvessels (Fig. 6d,e) indicating pericyte proliferation. Colocalization was also observed between PAL-E and PCNA (Fig. 6f). Serial sections stained with PCNA and PAL-E revealed that 14% of PAL-E positive microvessels showed evidence of PCNA immunostaining.

### Correlation of immunohistochemistry with clinical findings

We then correlated our immunohistochemical findings with clinical data (Table 2). Patients were classified according to four immunohistochemical criteria, as listed in Materials and methods.

**Figure 7:**
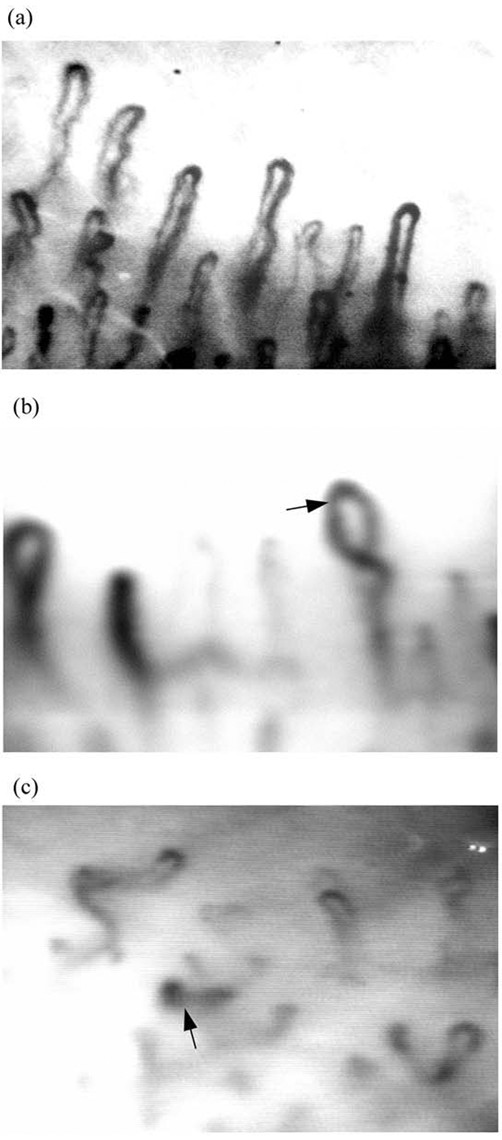
Nailfold capillaroscopy of **(a) **normal and **(b,c) **dcSSc patients. In the active pattern of capillary damage, frequent giant capillaries are present ((b) arrow) accompanied by moderate capillary loss and disorganisation of capillary architecture. Late disease pattern was characterized by severe capillary disorganisation with loss of capillaries ((c) arrow). Magnification ×150. dcSSc, diffuse cutaneous systemic sclerosis.

No significant association was found between mean disease duration (*p *= 0.11) and skin score (*p *= 0.97) and our immunohistochemical groups. We were able to assess the capillary patterns of eight of our ten dcSSc patients according to the criteria established by Cutolo *et al*. [35]. Of these eight patients, three had an active pattern of capillary damage while five displayed a late pattern of damage (Fig. 7). However, no significant association could be found between patterns of capillary damage and our immunohistochemical groups (*p *= 0.33).

## Discussion

The potential of pericytes as myofibroblast precursors in dcSSc merits investigation for a number of reasons. Firstly, a number of studies have highlighted the *in vitro *and *in vivo *capacity of pericytes to act as mesenchymal precursor cells [19,21,22,39]. Secondly, during liver and renal fibrosis, resident pericytes have been shown to differentiate into myofibroblasts. Finally, myofibroblasts have been previously reported in dcSSc skin [13,14], however, their function and origin remain unknown. The objective of our study was to investigate both the origin and biosynthetic profile of myofibroblasts in dcSSc.

In our current study, myofibroblasts were detected in dcSSc samples but were absent from normal skin. Correspondingly, increased expression of ED-A FN was detected only in those dcSSc samples containing myofibroblasts. Double-labelling experiments confirmed the expression of ED-A FN to interstitial myofibroblasts. To our knowledge, this is the first report of ED-A FN expression by myofibroblasts in dcSSc skin. ED-A FN was also found to be expressed by pericytes in the microvascular wall of dcSSc skin using double-labelling with α-SMA. Therefore, both myofibroblasts and pericytes appear to be key sources of ED-A FN in dcSSc skin. As ED-A FN is a pre-requisite for myofibroblast formation [9], the expression of ED-A FN by pericytes is likely to be of significance in the differentiation of perivascular fibroblasts and pericytes into myofibroblasts, and may represent a key step in linking microvascular damage and fibrosis.

An assessment of our immunohistochemical findings and clinical data revealed that the presence of myofibroblasts showed no significant association with either disease duration (*p *= 0.11) or skin score (*p *= 0.97). Additionally, no association was observed between the presence of myofibroblasts and either late or active capillary damage (*p *= 0.33). While our preliminary findings are based on a relatively small cohort of patients, we feel that further studies with a larger cohort of patients, designed to correlate immunohistochemical findings with clinical data on a patient-by-patient basis, may be highly informative.

Myofibroblasts and ED-A FN were found almost exclusively in the lower reticular dermis. A similar distribution of total fibronectin has also been observed in dcSSc skin [40]. The significance of this is unclear, however, it may reflect microenvironmental differences between the papillary and reticular dermis or the existence of heterogeneous fibroblast populations within the respective dermal compartments, or a combination of both these factors. Interestingly, it has been previously reported that reticular dermal fibroblasts are more inherently contractile in three-dimensional collagen matrices when compared with papillary dermal fibroblasts [41].

Our finding of myofibroblasts in six out of ten dcSSc patients contrasts slightly with two previous studies in which all dcSSc samples analysed contained myofibroblasts [13,14]. Discrepancies of this nature are unsurprising given the inherent heterogeneity across the scleroderma spectrum, differences in the staining protocols and the cross-sectional nature of these studies. However, it is worth reiterating that clear evidence of increased matrix biosynthesis was detected in eight out of ten dcSSc samples studied. Interestingly, only two samples contained both myofibroblasts and collagen-synthesizing cells. This corroborates two recent studies of murine lung fibrosis in which collagen-synthesizing cells were found to be distinct from α-SMA^+ve ^myofibroblasts [42,43] and a previous analysis of dcSSc skin, in which the presence of myofibroblasts did not correlate with α1(I) procollagen mRNA [14]. The relationship between myofibroblasts and the synthesis and deposition of fibrillar collagens is unknown and merits further investigation. Myofibroblasts and ED-A FN were not detected in skin taken from patients with atrophic dcSSc indicating that, as the disease progresses from the fibrotic to atrophic stage, myofibroblasts do not persist in the dermis.

Having recently been identified as a marker for cells with myofibroblastic potential, we also analysed the distribution of the Thy-1 antigen [18]. Two populations of Thy-1^+ve ^cells were identified in normal skin. Using double immunofluorescence labelling, we identified one population as pericytes, the second population, which was α-SMA^-ve ^and located interstitially, was identified as perivascular fibroblasts. In all dcSSc samples, Thy-1 was also found to be expressed by pericytes, however, there was a marked increase in Thy-1 immunostaining throughout the interstitium. Using double immunofluorescence labelling with α-SMA and ED-A FN antibodies, a number of the interstitial Thy-1^+ve ^cells were identified as myofibroblasts within the reticular dermis. However, Thy-1^+ve ^/EDA^-ve ^/SMA^-ve ^cells were also detected in the papillary dermis, suggesting that the Thy-1^+ve ^population can be divided into myofibroblastic and non-myofibroblastic populations depending on their location within the dermis.

**Figure 8:**
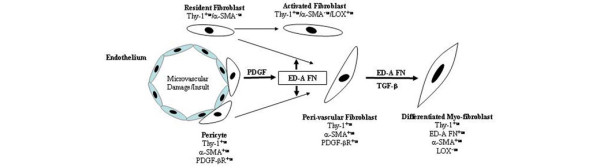
Convergence of microvascular pericytes and resident fibroblasts to a myofibroblast lineage in SSc. Two pathways potentially contribute to the fibrogenic response in dcSSc. Microvascular pericytes (Thy-1^+ve ^/α-SMA^+ve ^) become activated as a result of microvascular damage and produce the ED-A splice variant of fibronectin, a protein known to induce the myofibroblast phenotype. The microvascular derived ED-A FN in concert with the actions TGF-β may also act upon resident perivascular fibroblasts (Thy-1^+ve ^/α-SMA^-ve ^) stimulating their differentiation to myofibroblasts. Proliferation of both pericytes and fibroblasts may help to create a pool of potential myofibroblasts. α-SMA, alpha smooth muscle actin; dcSSc, diffuse cutaneous systemic sclerosis; ED-A FN, ED-A splice variant of fibronectin; TGF-β, transforming growth factor-beta.

Having demonstrated that in dcSSc skin, pericytes and myofibroblasts have an identical phenotype with respect to Thy-1, ED-A FN and α-SMA expression, we then hypothesized that a proliferation of pericytes may be in part responsible for the expansion of pericytes and generation of myofibroblasts in the interstitium. We found evidence of pericyte proliferation in two dcSSc cases containing myofibroblasts suggesting that any proliferative activity may be relatively short-lived. Increased pericyte proliferation and an increased pericyte to endothelial cell ratio have been recently reported in dcSSc skin [44] while in keloid skin, evidence of pericyte differentiation has also been observed [45]. Increased pericyte proliferation without a corresponding increase in capillary density has also been demonstrated in an *in vivo *tumour model and was found to be mediated by PDGF-β receptors [46]. PDGF is a potent mitogen and we have previously demonstrated that microvascular pericytes express PDGF-β receptors in dcSSc skin [4] suggesting that the observed pericyte proliferation in dcSSc skin may be in part mediated by the PDGF-β ligand/receptor axis. Our findings lead us to propose a hypothesis that would provide a cellular mechanism in dcSSc whereby initial microvascular damage could give rise to a fibrotic lesion through the increased production of ED-A FN by pericytes and perivascular fibroblasts, which, in concert with other factors (most notably TGF-β) would promote the differentiation of these cells into myofibroblasts (Fig. 8).

## Conclusion

We believe there is strong evidence to suggest that pericytes and myofibroblasts can be phenotypically linked by their mutual synthesis of ED-A FN in dcSSc and that this may represent an important pathway in the transition of a microvascular disease to a fibrotic one. We also suggest that pericytes represent an additional cell type that must be taken into account when considering pathogenic mechanisms and therapeutic targets in dcSSc.

## Abbreviations

α-SMA = alpha smooth muscle actin; DAPI = 4,6-diamidino-2-phenylindole; dcSSc = diffuse cutaneous systemic sclerosis; ED-A FN = ED-A fibronectin; FITC = fluorescein isothiocyanate; LOX = lysyl oxidase; PBS = phosphate buffered saline; PCNA = proliferating cell nuclear antigen; PDGF = platelet-derived growth factor; RNP = ribonuclear protein; TGF-β = transforming growth factor-beta.

## Competing interests

The authors declare that they have no competing interests.

## Authors' contributions

VSR was responsible for experimental work and analysis, drafting the manuscript and study design. KH carried out the nailfold capillaroscopy analysis. KC provided antisera and participated in drafting the manuscript. CPD provided the clinical data and analysis. CMB participated in drafting the manuscript. DJA contributed to study design, data analysis and drafting the manuscript.

## Figures and Tables

**Table 1:** Clinical and serological characteristics of SSc patients

Characteristics	Fibrotic (*n *= 10)	Atrophic (*n *= 6)
Mean age (range)	54 (39–72)	58 (37–69)
Mean disease duration, months (range)	11 (4–18)	96 (36–168)
Male/female	2/8	0/6
		
**Organ involvement**		
Mean skin score (range)	33 (19–41)	17 (11–24)
Oesophageal	7/10	3/6
Other gastrointestinal	4/10	1/6
Lung	4/10	2/6
Muscle	3/10	0/6
Renal	2/10	1/6
Cardiac	0/10	1/6
Pulmonary hypertension	2/10	0/6
		
**Serology**		
Antinuclear	10/10	6/6
Anti-topoisomerase 1	4/10	3/6
Anti-RNA polymerase I/III	2/10	1/6
Anti-nuclear RNP	1/10	1/6
		
**Microvascular damage**		
Structural capillary damage	10/10	6/6

RNP, ribonuclear protein; SSc, systemic sclerosis.

**Table 2:** Correlation of immunohistochemical and clinical data

	Duration (months)	Skin score	Capillary pattern	Collagen synthesis	Myofibroblasts
Patient 1	4	19	L	-	+++
Patient 2	6	24	A	+++	+
Patient 3	7	41	A	-	+++
Patient 4	9	38	N/D	-	+++
Patient 5	9	39	N/D	+++	+++
Patient 6	10	34	A	-	+++
Patient 7	11	40	L	+++	-
Patient 8	14	36	L	-	-
Patient 9	18	32	L	-	-
Patient 10	18	32	L	+++	+++

Immunohistochemistry is quantified as; -, absent, +, weak, +++, strong. Patterns of capillary damage are graded as A, active, L, late or N/D, not determined.

## References

[B1] Black CM (1995). The aetiopathogenesis of systemic sclerosis: thick skin-thin hypotheses. The Parkes Weber Lecture 1994. J R Coll Physicians Lond.

[B2] Prescott RJ, Freemont AJ, Jones CJ, Hoyland J, Fielding P (1992). Sequential dermal microvascular and perivascular changes in the development of scleroderma. J Pathol.

[B3] Sims DE (2000). Diversity within pericytes. Clin Exp Pharmacol Physiol.

[B4] Rajkumar VS, Sundberg C, Abraham DJ, Rubin K, Black CM (1999). Activation of microvascular pericytes in autoimmune Raynaud's phenomenon and systemic sclerosis. Arthritis Rheum.

[B5] Gabbiani G (2003). The myofibroblast in wound healing and fibrocontractive diseases. J Pathol.

[B6] Tomasek JJ, Gabbiani G, Hinz B, Chaponnier C, Brown RA (2002). Myofibroblasts and mechano-regulation of connective tissue remodelling. Nat Rev Mol Cell Biol.

[B7] Desmouliere A, Redard M, Darby I, Gabbiani G (1995). Apoptosis mediates the decrease in cellularity during the transition between granulation tissue and scar. Am J Pathol.

[B8] Zhang HY, Phan SH (1999). Inhibition of myofibroblast apoptosis by transforming growth factor beta(1). Am J Respir Cell Mol Biol.

[B9] Hinz B, Mastrangelo D, Iselin CE, Chaponnier C, Gabbiani G (2001). Mechanical tension controls granulation tissue contractile activity and myofibroblast differentiation. Am J Pathol.

[B10] Serini G, Bochaton-Piallat ML, Ropraz P, Geinoz A, Borsi L, Zardi L, Gabbiani G (1998). The fibronectin domain ED-A is crucial for myofibroblastic phenotype induction by transforming growth factor-beta1. J Cell Biol.

[B11] Odenthal M, Neubauer K, Meyer zum Buschenfelde KH, Ramadori G (1993). Localization and mRNA steady-state level of cellular fibronectin in rat liver undergoing a CCl4-induced acute damage or fibrosis. Biochim Biophys Acta.

[B12] Pujuguet P, Hammann A, Moutet M, Samuel JL, Martin F, Martin M (1996). Expression of fibronectin ED-A+ and ED-B+ isoforms by human and experimental colorectal cancer. Contribution of cancer cells and tumor-associated myofibroblasts. Am J Pathol.

[B13] Sappino AP, Masouye I, Saurat JH, Gabbiani G (1990). Smooth muscle differentiation in scleroderma fibroblastic cells. Am J Pathol.

[B14] Jelaska A, Korn JH (2000). Role of apoptosis and transforming growth factor beta1 in fibroblast selection and activation in systemic sclerosis. Arthritis Rheum.

[B15] Borrello MA, Phipps RP (1996). Differential Thy-1 expression by splenic fibroblasts defines functionally distinct subsets. Cell Immunol.

[B16] Silvera MR, Sempowski GD, Phipps RP (1994). Expression of TGF-beta isoforms by Thy-1+ and Thy-1- pulmonary fibroblast subsets: evidence for TGF-beta as a regulator of IL-1-dependent stimulation of IL-6. Lymphokine Cytokine Res.

[B17] Derdak S, Penney DP, Keng P, Felch ME, Brown D, Phipps RP (1992). Differential collagen and fibronectin production by Thy 1+ and Thy 1- lung fibroblast subpopulations. Am J Physiol.

[B18] Koumas L, Smith TJ, Feldon S, Blumberg N, Phipps RP (2003). Thy-1 expression in human fibroblast subsets defines myofibroblastic or lipofibroblastic phenotypes. Am J Pathol.

[B19] Schmitt-Graff A, Kruger S, Bochard F, Gabbiani G, Denk H (1991). Modulation of alpha smooth muscle actin and desmin expression in perisinusoidal cells of normal and diseased human livers. Am J Pathol.

[B20] Hattori M, Horita S, Yoshioka T, Yamaguchi Y, Kawaguchi H, Ito K (1997). Mesangial phenotypic changes associated with cellular lesions in primary focal segmental glomerulosclerosis. Am J Kidney Dis.

[B21] Doherty MJ, Ashton BA, Walsh S, Beresford JN, Grant ME, Canfield AE (1998). Vascular pericytes express osteogenic potential in vitro and in vivo. J Bone Miner Res.

[B22] Ivarsson M, Sundberg C, Farrokhnia N, Pertoft H, Rubin K, Gerdin B (1996). Recruitment of type I collagen producing cells from the microvasculature *in vitro*. Exp Cell Res.

[B23] Sundberg C, Ivarsson M, Gerdin B, Rubin K (1996). Pericytes as collagen-producing cells in excessive dermal scarring. Lab Invest.

[B24] LeRoy EC, Black C, Fleischmajer R, Jablonska S, Krieg T, Medsger TA, Rowell N, Wollheim F (1988). Scleroderma (systemic sclerosis): classification, subsets and pathogenesis. J Rheumatol.

[B25] Bombardieri S, Medsger TA, Silman AJ, Valentini G (2003). The assessment of the patient with systemic sclerosis. Introduction. Clin Exp Rheumatol.

[B26] Skalli O, Pelte MF, Peclet MC, Gabbiani G, Gugliotta P, Bussolati G, Ravazzola M, Orci L (1989). Alpha-smooth muscle actin, a differentiation marker of smooth muscle cells, is present in microfilamentous bundles of pericytes. J Histochem Cytochem.

[B27] Saalbach A, Kraft R, Herrmann K, Haustein UF, Anderegg U (1998). The monoclonal antibody AS02 recognizes a protein on human fibroblasts being highly homologous to Thy-1. Arch Dermatol Res.

[B28] Schlingemann RO, Dingjan GM, Emeis JJ, Blok J, Warnaar SO, Ruiter DJ (1985). Monoclonal antibody PAL-E specific for endothelium. Lab Invest.

[B29] Clarijs R, Schalkwijk L, Hofmann UB, Ruiter DJ, de Waal RM (2002). Induction of vascular endothelial growth factor receptor-3 expression on tumor microvasculature as a new progression marker in human cutaneous melanoma. Cancer Res.

[B30] Peters JH, Hynes RO (1996). Fibronectin isoform distribution in the mouse. I. The alternatively spliced EIIIB, EIIIA, and V segments show widespread codistribution in the developing mouse embryo. Cell Adhes Commun.

[B31] Li PA, He Q, Cao T, Yong G, Szauter KM, Fong KS, Karlsson J, Keep MF, Csiszar K. (2004). Up-regulation and altered distribution of lysyl oxidase in the central nervous system of mutant SOD1 transgenic mouse model of amyotrophic lateral sclerosis. Brain Res Mol Brain Res.

[B32] Smith-Mungo LI, Kagan HM (1998). Lysyl oxidase: properties, regulation and multiple functions in biology. Matrix Biol.

[B33] Chvapil M, McCarthy DW, Misiorowski RL, Madden JW, Peacock EE (1974). Activity and extractability of lysyl oxidase and collagen proteins in developing granuloma tissue. Proc Soc Exp Biol Med.

[B34] Kelman Z (1997). PCNA: structure, functions and interactions. Oncogene.

[B35] Cutolo M, Sulli A, Pizzorni C, Accardo S (2000). Nailfold videocapillaroscopy assessment of microvascular damage in systemic sclerosis. J Rheumatol.

[B36] Kobayashi H, Ishii M, Chanoki M, Yashiro N, Fushida H, Fukai K, Kono T, Hamada T, Wakasaki H, Ooshima A (1994). Immunohistochemical localization of lysyl oxidase in normal human skin. Br J Dermatol.

[B37] Hein S, Yamamoto SY, Okazaki K, Jourdan-LeSaux C, Csiszar K, Bryant-Greenwood GD (2001). Lysyl oxidases: expression in the fetal membranes and placenta. Placenta.

[B38] Saalbach A, Aneregg U, Bruns M, Schnabel E, Herrmann K, Haustein UF (1996). Novel fibroblast-specific monoclonal antibodies: properties and specificities. J Invest Dermatol.

[B39] Richardson RL, Hausman GJ, Campion DR (1982). Response of pericytes to thermal lesion in the inguinal fat pad of 10-day-old rats. Acta Anat (Basel).

[B40] Cooper SM, Keyser AJ, Beaulieu AD, Ruoslahti E, Nimni ME, Quismorio FP (1979). Increase in fibronectin in the deep dermis of involved skin in progressive systemic sclerosis. Arthritis Rheum.

[B41] Sorrell JM, Baber BA, Caplan AI (1996). Construction of a bilayered dermal equivalent containing human papillary and reticular dermal fibroblasts: use of fluorescent vital dyes. Tissue Engineering.

[B42] Hashimoto N, Jin H, Liu T, Chensue SW, Phan SH (2004). Bone marrow-derived progenitor cells in pulmonary fibrosis. J Clin Invest.

[B43] Lawson WE, Polosukhin VV, Zoia O, Stathopoulos GT, Han W, Plieth D, Loyd JE, Neilson EG, Blackwell TS (2005). Characterization of fibroblast-specific protein 1 in pulmonary fibrosis. Am J Respir Crit Care Med.

[B44] Helmbold P, Fiedler E, Fischer M, Marsch WC (2004). Hyperplasia of dermal microvascular pericytes in scleroderma. J Cutan Pathol.

[B45] Szulgit G, Rudolph R, Wandel A, Tenenhaus M, Panos R, Gardner H (2002). Alterations in fibroblast alpha1beta1 integrin collagen receptor expression in keloids and hypertrophic scars. J Invest Dermatol.

[B46] Furuhashi M, Sjoblom T, Abramsson A, Ellingsen J, Micke P, Li H, Bergsten-Folestad E, Eriksson U, Heuchel R, Betsholtz C (2004). Platelet-derived growth factor production by B16 melanoma cells leads to increased pericyte abundance in tumors and an associated increase in tumor growth rate. Cancer Res.

